# Immunosuppressive Environment of Pancreatic NENs—A Review

**DOI:** 10.3390/biomedicines14020366

**Published:** 2026-02-05

**Authors:** Jacek Kabut, Anita Gorzelak-Magiera, Jakub Sokołowski, Wiktoria Żelazna, Mateusz Stępień, Marta Strauchman, Natalia Jaworska, Beata Kos-Kudła, Iwona Gisterek-Grocholska

**Affiliations:** 1 Department of Oncology and Radiotherapy, Medical University of Silesia, 40-615 Katowice, Poland; anitagor@op.pl (A.G.-M.); igisterek@sum.edu.pl (I.G.-G.); 2 Student Scientific Club, Department and Clinic of Oncology and Radiotherapy, The Faculty of Medical Sciences, Medical University of Silesia, 40-615 Katowice, Polandwikzel575@gmail.com (W.Ż.); matthew.x.stepien@gmail.com (M.S.); poczta.martys@gmail.com (M.S.);; 3 Department of Endocrinology and Neuroendocrine Tumours, Department of Pathophysiology and Endocrinology, Faculty of Medical Sciences in Zabrze, Medical University of Silesia, Katowice, ul. Ceglana 35, 40-514 Katowice, Poland; beatakos@ka.onet.pl

**Keywords:** pancreatic neuroendocrine tumors, immunotherapy, antiangiogenic therapy, immune checkpoints, tumor microenvironment, immunosuppression

## Abstract

Pancreatic neuroendocrine neoplasms (pNENs) are rare tumors with significant biological diversity. Despite significant improvements in diagnostics and a growing range of available therapies, long-term disease control remains difficult in advanced cases. The tumor microenvironment, which in pNENs adopts a predominantly immunosuppressive profile and promotes tumor development, is attracting increasing attention. A complex network of interactions dominates the tumor tissue, including M2 macrophages, regulatory T cells, and numerous pathways that inhibit effector lymphocyte activity. M2 macrophages, through the secretion of anti-inflammatory cytokines and exosome-mediated signaling, support angiogenesis while simultaneously attenuating the cytotoxic response. Simultaneously, receptors and ligands associated with immune checkpoints are overexpressed. In addition to classic molecules such as PD-1/PD-L1 and CTLA-4, the role of B7x and CD276 is increasingly being emphasized, as their presence correlates with rapid disease progression and poor prognosis. To date, attempts to use checkpoint inhibitors as monotherapy have yielded modest clinical benefits. However, approaches based on combination strategies—both in the form of dual immune blockade and in combination with chemotherapy or angiogenesis-targeted therapy—have shown significantly greater activity. Therapies using tyrosine kinase inhibitors, such as sunitinib and newer drugs (lenvatinib, surufatinib, cabozantinib), may partially normalize the tumor’s disrupted vascular architecture and thus increase its susceptibility to immunological interventions. In the coming years, it will be crucial not only to overcome the immunosuppressive nature of the TME but also to identify predictive biomarkers that will allow for more precise patient selection. This approach may open the way to more effective, personalized therapies for pNENs.

## 1. Introduction

Pancreatic neuroendocrine neoplasms (pNENs) constitute a group of rare tumors originating from cells of the pancreatic neuroendocrine system, diverse in terms of morphology, prognosis, and clinical behavior. Their phenotype reflects the complex biology of neuroendocrine cells, which translates into a wide spectrum of clinical presentations—from mildly symptomatic lesions to aggressive, rapidly growing tumors [1].

**Figure 1:**
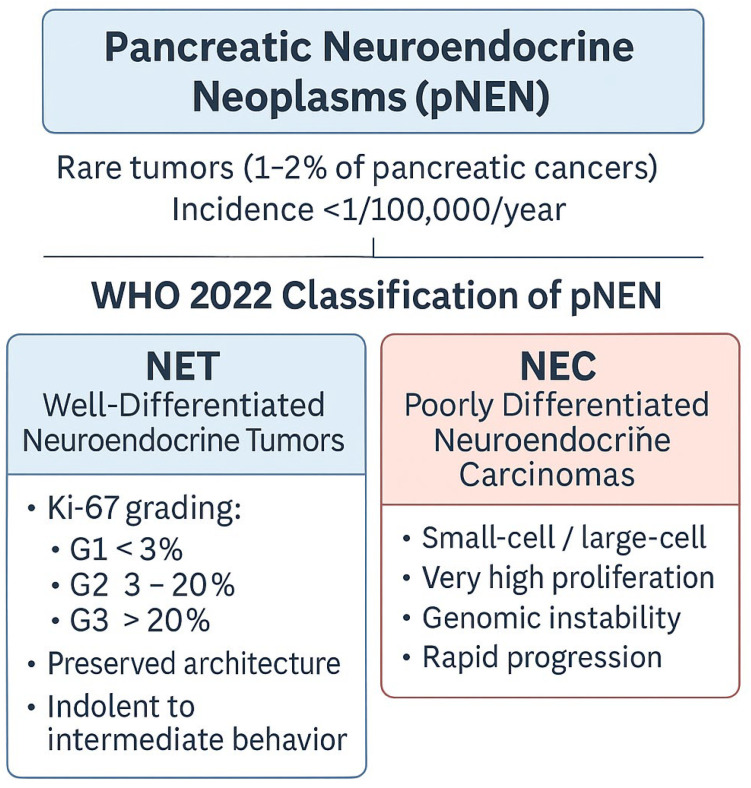
Overview of pancreatic neuroendocrine neoplasms (pNENs).

Pancreatic neuroendocrine neoplasms constitute only 1–2% of all pancreatic neoplasms, and their incidence remains very low, estimated at less than 1 case per 100,000 people per year [2]. Most of them, nearly 90%, are classified as pNETs. These tumors are most often diagnosed in patients over 60 years of age. Although sporadic forms predominate, approximately 10% of cases are associated with genetically determined conditions, such as polyadenomatosis syndromes or other rare germline mutations [3] [Figure 1].

In the entire pNEN group, the median survival is approximately 136 months. In the presence of lymph node involvement, it is shortened to approximately 77 months, while in the presence of distant metastases, it drops to nearly 24 months [4].

The current WHO 2022 classification, which encompasses neuroendocrine tumors of the gastrointestinal tract and pancreatobiliary system, distinguishes two fundamental categories: well-differentiated neuroendocrine tumors (NETs) and poorly differentiated neuroendocrine carcinomas (NECs). NETs constitute the largest subgroup and are further stratified into three grades—G1, G2, and G3—based primarily on the Ki-67 proliferative index. Despite increasing proliferative activity across grades, well-differentiated NETs largely preserve the architectural and cytological features of neuroendocrine neoplasms, and their clinical course is generally more indolent compared with poorly differentiated tumors [5].

Importantly, the recognition of pNET G3 as a well-differentiated neoplasm with high proliferative activity has highlighted the marked biological heterogeneity within high-grade pancreatic neuroendocrine neoplasms. Growing evidence indicates that pNET G3 differs substantially from pNECs with respect to genomic alterations, tumor growth dynamics, treatment sensitivity, and clinical outcomes, supporting its classification as a distinct biological and clinical entity rather than an intermediate form between low-grade pNETs and NECs [5].

In contrast, poorly differentiated NECs, including small cell and large cell subtypes, represent a biologically aggressive category characterized by extremely high proliferative rates, pronounced genomic instability, and early systemic dissemination. These tumors frequently harbor molecular alterations typical of high-grade epithelial malignancies and exhibit a substantial loss of neuroendocrine differentiation. Although classical neuroendocrine markers may remain detectable by immunohistochemistry, their expression is often heterogeneous or patchy, reflecting the dedifferentiated nature of these neoplasms [5,6].

Crucially, these histological and molecular distinctions are accompanied by distinct immune landscapes. Well-differentiated pNETs are typically characterized by low immune cell infiltration and a predominantly immunosuppressive tumor microenvironment, consistent with an immune-cold or immune-excluded phenotype. In contrast, poorly differentiated pNECs more often exhibit features of immune-inflamed (“immune-hot”) tumors, including higher densities of tumor-infiltrating immune cells and increased expression of immune checkpoint molecules. These differences provide a biological rationale for addressing tumor microenvironment–driven mechanisms of immune regulation and therapeutic vulnerability separately in pNETs and pNECs in the following sections [7].

Hormonal function also remains a crucial element in the classification of pNENs. Some tumors remain hormonally inactive and only become apparent in advanced stages, often as a result of incidental imaging findings. Active forms, however, produce excess levels of specific hormones (including insulin, gastrin, glucagon, or VIP), manifest through characteristic clinical syndromes that can significantly impact the course of the disease and treatment [2,8].

Such wide morphological and biological diversity has direct clinical significance: it determines the choice of therapy, influences prognosis, and forms the basis for decisions regarding systemic treatment. Understanding these differences is essential both in everyday clinical practice and in designing new therapeutic strategies.

**Figure 2:**
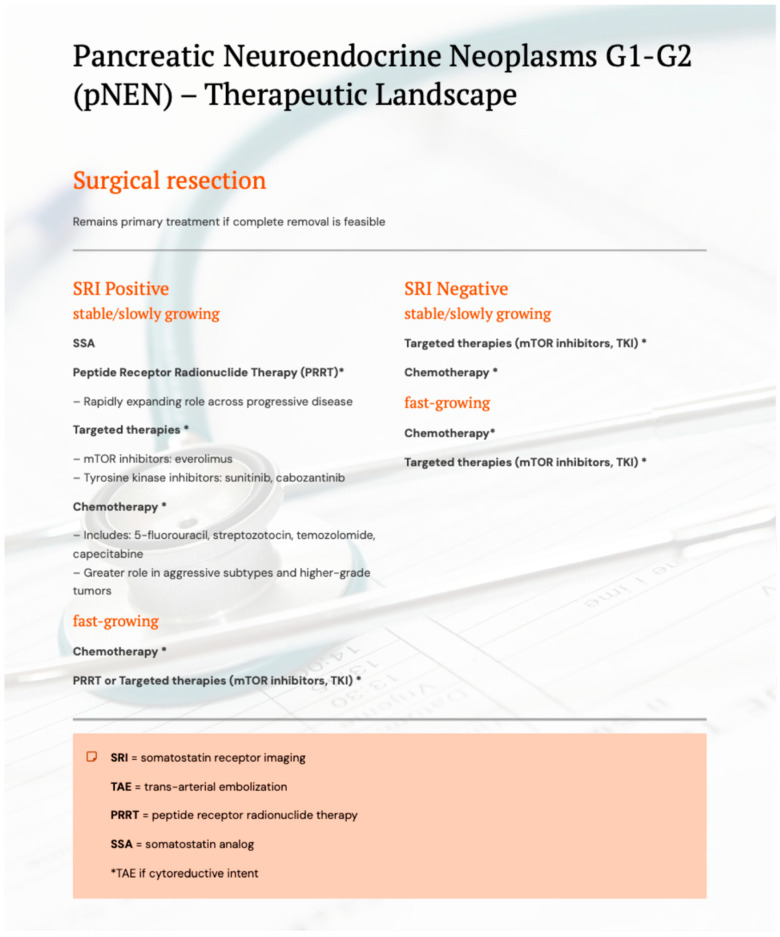
Therapeutic landscape of pancreatic neuroendocrine neoplasms (pNENs).

Peptide receptor radionuclide therapy (PRRT) is also gaining increasing importance. Treatment with ^177Lu-Dotatate has shown promising results, especially in patients with high somatostatin receptor expression on imaging studies, offering extended time to progression and improved disease control [4,6]. Treatment options for pNENs are presented in Figure 2. An integrated therapeutic approach, taking into account the degree of malignancy, somatostatin receptor expression and tumour growth dynamics, is reflected in the treatment algorithm presented in Figure 2, developed on the basis of the current recommendations of the European Neuroendocrine Tumor Society (ENETS) and referring to the therapeutic management of patients with pNEN with malignancy grades G1 and G2. In the case of tumours with a higher degree of malignancy, including pNEN G3 and NEC, the standard of care is to start systemic treatment with chemotherapy [9].

The primary treatment method for pancreatic neuroendocrine tumors remains surgical resection, provided that complete tumor removal is feasible. In hormonally active lesions and in patients with unresectable or metastatic disease, somatostatin analogues play a key role by controlling hormone-related symptoms and slowing tumor progression [10].

However, a subset of patients eventually develops resistance to somatostatin analogue therapy, necessitating alternative systemic approaches. In such cases, molecularly targeted agents, including mTOR inhibitors (e.g., everolimus) and tyrosine kinase inhibitors—most notably sunitinib—are commonly employed. Chemotherapy regimens, such as 5-fluorouracil, streptozotocin, temozolomide, or capecitabine, may be used as alternative or complementary strategies, particularly in tumors with higher proliferative activity [4].

Peptide receptor radionuclide therapy (PRRT) has emerged as an increasingly important therapeutic option in patients with high somatostatin receptor expression demonstrated on functional imaging. Treatment with ^177Lu-DOTATATE has been associated with prolonged progression-free survival and improved disease control, particularly in well-differentiated pNETs [4,6]. Importantly, high-grade pancreatic neuroendocrine neoplasms should not be regarded as a uniform therapeutic category. While poorly differentiated neuroendocrine carcinomas (NECs) are typically managed with systemic chemotherapy as the standard of care, well-differentiated PanNET G3 represents a distinct biological entity with a broader therapeutic spectrum. In selected patients with PanNET G3—especially those with preserved somatostatin receptor expression and less aggressive tumor kinetics—PRRT may constitute a valuable treatment option, either as monotherapy or as part of a sequential treatment strategy [11]

Recent prospective evidence indicates that contemporary systemic therapies in poorly differentiated pancreatic neuroendocrine carcinomas provide limited but measurable clinical benefit, underscoring the aggressive biology of pNECs and the persistent unmet need for optimized treatment strategies [12].

An integrated therapeutic approach that incorporates tumor grade, somatostatin receptor status, and tumor growth dynamics is reflected in the treatment algorithm presented in Figure 2. This algorithm is based on current European Neuroendocrine Tumor Society (ENETS) recommendations (2023) and primarily refers to the management of patients with well-differentiated pNETs G1–G2, while explicitly acknowledging the need for individualized treatment decisions in PanNET G3 and the distinct role of chemotherapy in poorly differentiated NECs [9].

In recent years, increasing attention has been paid to the role of the tumor microenvironment (TME) as a potential therapeutic target in pNENs. The TME comprises a complex network of non-cellular components—such as the extracellular matrix (ECM), cytokines, chemokines, growth factors, and extracellular vesicles—as well as a variety of non-neoplastic cells present within the tumor. These include fibroblasts, epithelial cells, adipocytes, neurons, and numerous immune cell populations that, to varying degrees, influence tumor behavior [13].

TME elements support tumor cell survival, facilitate local invasion and dissemination, and participate in angiogenesis and the regulation of tumor metabolism. The immune infiltrate of pNENs is observed to be highly heterogeneous—immune cells can exhibit both anti-neoplastic and disease-promoting properties [14].

Importantly, recent preclinical studies in murine tumor models indicate that immunotherapy resistance may not solely result from the absence of immune cells, but rather from functional suppression within the TME. Tichet et al. demonstrated that targeted delivery of an engineered IL-2 variant to PD-1-expressing T cells using a bispecific PD1-IL2v immunocytokine promotes the expansion of stem-like, tumor-reactive CD8^+^ T cells, while concomitant PD-L1 blockade reprograms tumor-associated macrophages and tumor vasculature toward a pro-inflammatory, antigen-presenting phenotype, effectively overcoming intrinsic resistance to immunotherapy in pancreatic neuroendocrine tumor models [15]. These findings highlight the critical interplay between T-cell stemness and myeloid cell plasticity within the TME and underscore the therapeutic potential of strategies aimed at immune microenvironment remodeling.

Understanding the mechanisms responsible for immunosuppression in the TME and its pharmacological reversal therefore represents a promising strategy that may improve the effectiveness of systemic therapies used in pNENs in the future.

## 2. Objective

The aim of this paper is to review the current literature on the microenvironment of neuroendocrine tumors, with particular emphasis on the role of immune cells and other microarray components, and to discuss the importance of expression of classical and novel immune checkpoints—including PD-1/PD-L1, CTLA-4, B7x, and CD276—in the context of immune modulation and potential therapeutic strategies.

## 3. Search Strategy

A literature search on pathogenic mechanisms and clinical implications regarding tumor immunology microenvironment in pNEN was conducted using the PubMed, Scopus, and Google Scholar databases. Relevant keywords and associated phrases were applied: “T cells”, “M2 macrophages”, “MDSC stem cells”, “TIM-3”, “LAG-3”, “BD-1”, “immunotherapy in pancreatic neuroendocrine tumor/pancreatic neuroendocrine neoplasms/pNET/pNEN”. The literature search was conducted between September and November 2025. The language of the studies was primarily English. Studies were published within the period from 2005 to 2025.

The types of studies analyzed included original articles, systematic reviews, and meta-analyses. Studies were excluded if they were published in languages other than English or were case reports, letters or editorials. Article selection was performed in stages: initial screening by title and abstract, followed by full-text evaluation of potentially eligible studies.

In order to prepare a summary of clinical trials involving immunotherapy and antiangiogenic drugs, the following databases were searched: ClinicalTrials.gov (https://clinicaltrials.gov/ access date: 15 December 2025), WHO International Clinical Trials Registry Platform (ICTRP), and the EU Clinical Trials Register. The search combined the terms “pancreatic neuroendocrine tumor,” “pancreatic neuroendocrine neoplasm” and “pNET” with the names of known immunotherapeutic agents: pembrolizumab, toripalimab, cemiplimab, dostarlimab, spartalizumab, nivolumab, avelumab, durvalumab, atezolizumab, tremelimumab, ipilimumab, relatlimab fianlimab (REGN3767), sabatolimab (MBG453), tiragolumab, vibostolimab, ociperlimab, magrolimab (Hu5F9-G4), urelumab, utomilumab, MOXR0916, bintrasfusp alfa (M7824), IFN-α, and antiangiogenic drugs: nintedanib, bevacizumab, pertuzumab, sunitinib, sulfatinib, pazopanib, lenvatynib.

### 3.1. Immune Cells of pNEN Microenvironment

The tumor microenvironment (TME) in pancreatic and gastrointestinal neuroendocrine neoplasms (GEP-NENs) is characterized by a complex and rich cellular composition, encompassing numerous immune cell populations, such as T and B lymphocytes, natural killer (NK) cells, monocytes, tumor-associated macrophages (TAMs), mast cells, fibroblasts, and myeloid-derived suppressor cells (MDSCs). The presence of these populations promotes the creation of an immunosuppressive environment that supports tumor progression and enables it to evade immune surveillance [7].

The immune infiltration profile in neuroendocrine neoplasms is characterized by significant heterogeneity, both between tumors originating from different organs and within the same primary site [7]. This complexity was confirmed in a study by Yo Zhou et al., in which the transcriptome of 24,544 tumor cells was analyzed using single-cell RNA sequencing (scRNA-seq), identifying nine major cell clusters, including epithelial cells, endothelial cells, fibroblasts, B lymphocytes, monocytes, macrophages, mast cells, NK cells, and T lymphocytes. The authors also demonstrated significant differences in the composition of the microenvironment between the primary tumor and metastatic lesions, as well as the presence of cell subpopulations characteristic of specific anatomical locations [16].

Depending on the dominant immune cell populations, neuroendocrine tumors may exhibit characteristics of so-called “hot” tumors, characterized by an intense inflammatory infiltrate, or “cold” tumors, characterized by a limited immune response [16,17]. Neuroendocrine carcinomas (NECs) typically exhibit an immunologically “hot” phenotype, with high numbers of tumor-infiltrating lymphocytes (TILs), whereas well-differentiated neuroendocrine tumors (NETs) more often exhibit a “cold” microenvironment, deficient in TILs. The infiltration of CD3+ and CD8+ lymphocytes and CD68+ or CD163+ macrophages is more pronounced in pNECs than in pNETs [7,16,17].

pNETs remain structures with high metabolic activity and the ability to modulate the immune response by secreting numerous immunosuppressive cytokines, such as TGF-β, IL-10, and IL-6. These factors play a significant role in the recruitment of MDSCs and tumor-associated macrophages, particularly the CD68+ subpopulation and M2-like macrophages. As a consequence, the activity of effector T lymphocytes is inhibited and the mechanisms of immunosuppression mediated by regulatory T cells (Treg) are intensified, which promotes the progression of cancer [18].

### 3.2. T Lymphocytes

The presence of T lymphocytes in neuroendocrine tumors has been confirmed both by direct identification of various subpopulations of these cells in pNETs and in indirect studies, which demonstrated elevated levels of interleukin 2 (IL-2), a cytokine produced primarily by activated CD4+ and CD8+ T lymphocytes [19,20]. These data indicate a significant role of the T cell-dependent response in shaping the immune microenvironment of pNETs.

In the study by Silva et al., T cell infiltration in intra- and peritumoral compartments was assessed using a panel of markers including CD3 (a general T cell marker), CD45RO (memory T cells), CD8 (cytotoxic T cells), and FOXP3 (regulatory T cells). High levels of intratumoral T cell infiltration were observed in 32–65% of pNETs, with similar results obtained for the peritumoral compartment. Furthermore, pancreatic neuroendocrine tumors demonstrated significantly greater lymphocytic infiltration compared to small intestinal NETs in terms of CD3, CD45RO, and CD8 expression, with a concomitant low proportion of FOXP3+ regulatory cells in both tumor locations [21]. Similar observations were reported by Ryschich et al., who demonstrated increased intratumoral infiltration of CD3+, CD4+, and CD8+ T lymphocytes, which was interpreted as a result of an active T-cell-dependent immune response [22].

The prognostic significance of lymphocytic infiltration was emphasized in the study by Katz et al., which demonstrated that high peritumoral CD3+ T lymphocyte levels predicted longer recurrence-free survival (RFS) in patients with pNETs. Analysis of intermediate-grade tumors showed that patients with a high CD3+ T cell infiltrate achieved a median RFS of 128 months, compared with 61 months in patients with a low level of intratumoral infiltrate. Concurrently, a low percentage of regulatory T cells (FOXP3+) in liver metastases of NETs was associated with prolonged survival, suggesting a negative role for this subpopulation in the disease course [23]. Similarly, De Reuver et al. indicated that the presence of FOXP3+ regulatory T cells may reflect immunosuppressive mechanisms in pNETs and contribute to tumor progression [24].

Additional information regarding the importance of lymphocytic infiltration was provided by Bösch et al., who demonstrated that a high level of tumor-infiltrating lymphocytes (TILs), coexisting with increased PD-1 receptor expression, correlated with shorter overall survival and higher histological grade of GEP-NENs. In the analyzed cohort, as many as 50% of G3 tumors were characterized by high levels of TILs, compared with 17.1% of G1/2 tumors (*p* < 0.001). Importantly, patients with low-grade lymphocytic infiltration achieved a significantly longer median overall survival (53.9 months) compared to patients with high TIL counts (39.4 months; *p* < 0.001) [25].

### 3.3. Macrophages

Tumor-associated macrophages (TAMs) constitute one of the key immune cell populations present in the tumor microenvironment. They are characterized by significant phenotypic and functional heterogeneity, reflecting their ability to adapt to local tumor conditions. Numerous cancer models have demonstrated that TAMs participate in disease progression at multiple levels, including by promoting genetic instability, creating niches favoring cancer stem cell survival, and facilitating invasion and metastasis [26].

Classically, macrophages are divided into M1 and M2 phenotypes. M1 macrophages, activated under proinflammatory conditions, participate in the antimicrobial response and exhibit antitumor properties. M2 macrophages, induced in an anti-inflammatory environment, primarily perform immunoregulatory and tissue repair functions. However, the tumor microenvironment is dominated by macrophages with characteristics similar to the M2 phenotype, which inhibit an effective immune response against tumor cells. Myeloid-derived suppressor cells (MDSCs), which are closely related to TAMs and, in some cases, constitute their precursors, also play a significant role in this process [27].

Macrophages polarized toward the M2 phenotype demonstrate the ability to intensely secrete biological mediators, including cytokines and growth factors such as TGF-β, TNF-α, IL-1β, IL-8, CCL2, CCL5, ERO-1, matrix metalloproteinases (MMP-2, MMP-3, MMP-9), CSF-1, VEGF, and PLGF. These substances play a significant role in remodeling the tumor microenvironment, enhancing angiogenesis, and increasing the metastatic potential of tumor cells [28,29,30].

The clinical significance of this activity was confirmed, among others, in a study by Boemi et al., in which plasma concentrations of IP-10, MCP-1, and IL-8 were significantly higher in patients with pancreatic neuroendocrine tumors compared to healthy individuals. Importantly, high IP-10 expression correlated with increased T cell infiltration in the tumor microenvironment. MCP-1, in turn, through interaction with the CCR2 receptor, participates in the recruitment of both tumor-associated macrophages and myeloid-derived suppressor cells, thus enhancing the immunosuppressive nature of the tumor microenvironment [31].

### 3.4. The Significance of Tumor-Associated Macrophages in pNETs

**Figure 3:**
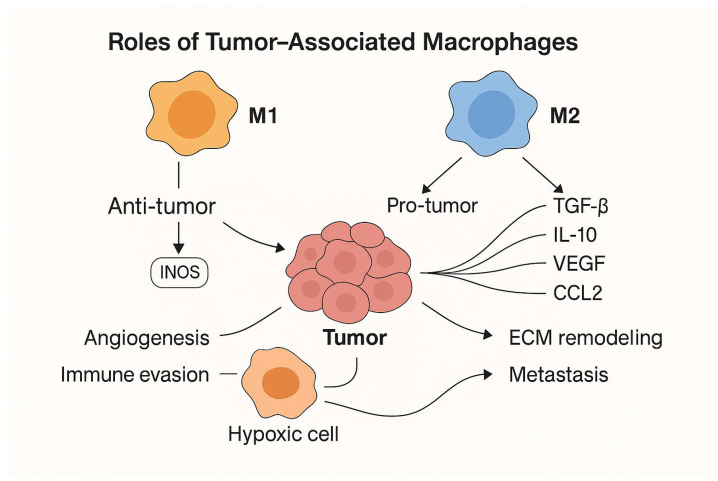
Roles of tumor-associated macrophages (TAMs) in the tumor microenvironment. M1-polarized macrophages exert antitumor activity through proinflammatory mechanisms, including inducible nitric oxide synthase (iNOS) expression, whereas M2-polarized macrophages promote tumor progression by secreting immunosuppressive and proangiogenic factors (e.g., TGF-β, IL-10, VEGF, CCL2), leading to extracellular matrix remodeling, angiogenesis, and metastasis.

Tumor-associated macrophages (TAMs) exhibit significant phenotypic heterogeneity. However, in many malignancies, the dominant fraction is composed of M2 cells, characterized by the expression of markers such as CD163. In pancreatic neuroendocrine neoplasms, an increased number of this subpopulation correlates with the presence of metastases to regional lymph nodes, lymphatic vessel invasion, and neural involvement. These phenomena translate into shorter disease-free survival, higher histopathological grade, and a higher incidence of distant metastases [32,33] [Figure 3].

Similar observations were reported in a study by Zhijiang Chen et al., which demonstrated a significant association between the intensity of CD163+ macrophage infiltration and disease progression and the ability to metastasize in a group of pan-NENs. The authors also confirmed a significantly higher accumulation of TAMs in pancreatic neuroendocrine carcinomas (pNECs) compared to well-differentiated neuroendocrine tumors (pNETs). In addition to CD163, CD68 expression is also considered a significant marker of M2 macrophages [34].

Patients with AJCC stage III or IV clinical advancement were shown to have a significantly higher degree of CD68+ macrophage infiltration (*p* = 0.042). Comparative analyses also revealed a significantly higher number of these cells in metastatic foci in the liver compared to primary tumors (*p* = 0.0069). An increased presence of TAMs was also associated with a higher risk of disease recurrence in patients with non-functional pNENs, compared to patients with a lower degree of CD68+ infiltration [35]. In the study by Iris H. Wei et al., a high CD68 index was identified as one of the factors associated with disease recurrence after surgical treatment [32]. Cai et al., in turn, demonstrated that the presence of double-positive CD68+/CD163+ macrophages correlated with shorter disease-free survival (DFS) and disease-specific survival (DSS) in a multivariate analysis [36].

Contrary results were presented in the Takahashi study, in which type II macrophages proved to be a significant prognostic factor for recurrence-free survival (RFS), while overall survival (OS) was more strongly associated with tumor grade and high PD-1 expression on T lymphocytes. Importantly, the presence of T lymphocytes with the PD-1^high phenotype and type II macrophages exhibiting high PD-L1 expression were associated with an unfavorable prognosis in patients with pNENs [7]. However, these results are partially inconsistent with the observations of Imam et al., who did not show significant differences in the number of CD163+ cells between patients with relapse and/or death and those without these events [32].

### 3.5. Polarisation of Macrophages in pNEN Microenvironment

The mechanisms underlying macrophage polarization in the pNEN microenvironment were analyzed in detail in a study by Feiyu et al., which demonstrated significant overexpression of miR-4488 in exosomes secreted by hypoxic pNEN cells. This microRNA, once internalized by macrophages, leads to the suppression of RTN3 protein, which results in increased fatty acid oxidation and activation of the PI3K/AKT/mTOR signaling pathway by inhibiting FABP5 expression [29]. The process of polarization toward the M2 phenotype is further enhanced by the CEACAM5 protein, present at high concentrations in pNEN exosomes, which induces this process via the MAPK pathway [37].

Macrophages polarized to the M2 phenotype play a significant role in facilitating pNEN metastasis, particularly to the liver—the most common site of disease dissemination [38]. One key mechanism is the induction of matrix metalloproteinase (MMP2) expression, which leads to a positive feedback loop involving the miR-4488/RTN3/FABP5/MMP2 axis in cancer cells. This phenomenon promotes increased invasiveness and disease progression. At the same time, M2 macrophages are characterized by increased expression of markers such as CD163, IL-10, VEGFA, and TGF-β, while simultaneously inhibiting the expression of markers characteristic of the M1 phenotype, including TNF-α and iNOS, which promotes angiogenesis and metastasis [29].

### 3.6. Other Molecular Pathways and Mechanisms of T Cell Suppression

An increasing body of evidence indicates that M2 macrophages play a key role in shaping the immunosuppressive microenvironment of pNENs by modulating the PD-1/PD-L1 axis. A study by Chen et al. demonstrated a significant correlation between the expression of M2 macrophage markers and PD-L1 levels in pNEN tumor tissue, suggesting that these cells may be a major source of PD-L1 ligand within the TME. Consequently, TAMs may mediate the inhibition of effector T cell activity and promote tumor immune escape [34].

Additional mechanisms of immunosuppression were described in a study by Zhong et al., which demonstrated elevated piR-hsa-30937 expression in small extracellular vesicles (sEVs) isolated from the serum of pNEN patients. This microRNA led to activation of the AKT pathway by directly affecting the PTEN gene, resulting in increased expression of CD276 on the surface of macrophages. Activated CD276+ macrophages demonstrated the ability to inhibit T cell proliferation and limit interferon-gamma production, which was associated with an unfavorable clinical prognosis [39].

Another important regulator of the tumor microenvironment is miR-183-5p, whose presence was demonstrated in sEV vesicles derived from pNEN cells. By activating the PDCD4/PI3K/AKT/mTOR axis, this microRNA induced overexpression of SPP1 and promoted macrophage polarization toward the M2 phenotype. Importantly, sEV-miR-183-5p secretion was controlled by TP53 gene mutations, and high plasma concentrations of this microRNA correlated with higher tumor malignancy and clinical progression of the disease [40]. The impact of therapy on the pNEN microenvironment was assessed, among others, in a study by Schiavo Lena et al., which analyzed immunological changes in patients undergoing somatostatin receptor-targeted radioisotope therapy (PRRT). The authors observed an increased density of CD163+ macrophages with an M2 phenotype in tumors following PRRT treatment. Furthermore, these cells tended to adopt a morphology resembling epithelial cells, in contrast to the dendritic macrophages observed in the control group. However, it should be emphasized that the study included a small number of patients, and none of the analyzed parameters demonstrated a significant association with prognosis. An additional limitation was the relatively short median follow-up [41].

Taken together, accumulating evidence indicates that macrophage activity varies substantially across subtypes of pancreatic neuroendocrine neoplasms. In well-differentiated pNETs (G1–G2), the tumor microenvironment is typically enriched in immunosuppressive, M2-like tumor-associated macrophages, which promote angiogenesis and facilitate immune escape. In pNET G3, macrophage polarization appears more heterogeneous, likely reflecting higher proliferative rates and increased immune pressure within the tumor microenvironment. By contrast, poorly differentiated pNECs are more frequently associated with an immune-inflamed microenvironment, characterized by abundant and transcriptionally active macrophages with enhanced antigen-presenting features and increased PD-L1 expression. This immune contexture supports the concept of pNECs as immune-hot tumors and underscores fundamental differences in macrophage-driven immune regulation across pNEN subtypes [33,42].

### 3.7. NK Cells

Natural killer (NK) cells constitute an important element of the antitumor response due to their ability to directly destroy tumor cells regardless of antigen presentation [43]. However, similarly to T lymphocytes, NK cells in pNENs exhibit features of immune exhaustion, manifested by overexpression of checkpoint receptors such as LAG-3, TIM-3, and TIGIT, which limits their cytotoxic activity [44]. Clinical studies have shown that NK cell activity in the peripheral blood of patients with neuroendocrine tumors, including pancreatic tumors, was significantly reduced compared to healthy individuals (*p* < 0.05) and patients with gastrinoma (*p* < 0.02) [45].

### 3.8. Mast Cells

Mast cells (MCs) are ubiquitously present in the pNEN microenvironment and are identified primarily by the expression of the marker CD117 (c-Kit). A study by Mo et al. demonstrated that a high density of mast cells within the tumor independently predicted longer progression-free survival (PFS), while a low number of MCs correlated with higher malignancy grade and the presence of distant metastases. These results suggest a potentially protective role of mast cells in pNENs [46].

### 3.9. Dendritic Cells

Dendritic cells (DCs) are among the most effective antigen-presenting cells and play a key role in initiating the immune response. The process of DC maturation, which involves the transition from an immature to a mature phenotype, is essential for effective T cell activation. A study by Young et al. demonstrated that increased numbers of DCs in PanNETs were associated with upregulation of checkpoint genes and an increase in the Treg cell population, which correlated with shorter overall patient survival [47]. Meng et al. observed a reduced level of immature DCs infiltration in metastatic tumors, suggesting that these cells may contribute to limiting the infiltration of other immune cells by creating an immunosuppressive microenvironment favoring disease progression [38].

### 3.10. Immune Checkpoints

Immune checkpoints (ICs) are key regulatory elements of the immune response, located primarily on the surface of immune cells. The physiological role of PD-1 is to limit excessive activation of the immune system during inflammatory reactions and to prevent autoimmune reactions [48].

**Figure 4:**
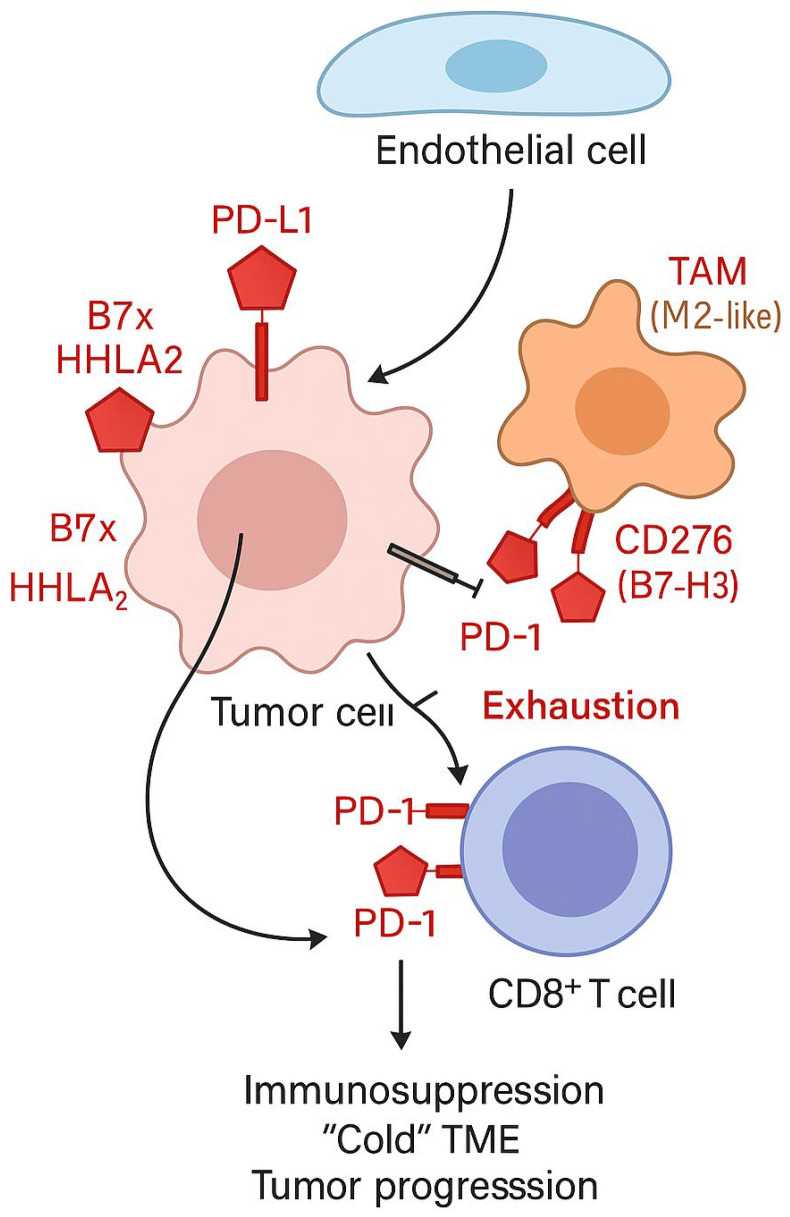
Immune checkpoint regulation in the tumor microenvironment (TME) of pancreatic neuroendocrine tumors (pNETs). Tumor cells, tumor-associated macrophages (TAMs, M2-like), and endothelial cells contribute to immune evasion through the expression of immune checkpoint ligands, including PD-L1, CD276 (B7-H3), B7x, and HHLA2. PD-L1 expressed on tumor and endothelial cells engages PD-1 receptors on CD8^+^ T cells, leading to T-cell exhaustion and reduced antitumor activity. TAM-derived CD276 further suppresses T-cell function and reinforces an immunosuppressive, “cold” tumor microenvironment.

Their primary function is to modulate lymphocyte activity in response to antigen presentation through inhibitory or activating signals. In most cases, these are T cell surface receptors and their ligands, which collectively control the intensity and duration of the immune response. One of the primary mechanisms of tumor escape from immune surveillance is the deregulation of the expression of ICs, which allows tumor cells to evade elimination despite the presence of tumor antigens [49] [Figure 4].

The discovery of CTLA-4 inhibitors on T lymphocytes led to the development of ipilimumab, the first CTLA-4-blocking antibody, which was approved by the Food and Drug Administration (FDA) for the treatment of melanoma in 2011. Following the undisputed success of ipilimumab, another checkpoint protein was discovered: programmed cell death protein 1 (PD-1). The first FDA-approved PD-1 inhibitor was nivolumab. Subsequently, other PD-1 and programmed cell death ligand 1 (PD-L1) inhibitors were approved, such as pembrolizumab, atezolizumab, durvalumab, and avelumab. Although ICIs have significantly improved treatment outcomes in solid tumors, only 20–40% of patients respond to treatment [49,50].

### 3.11. Expression and Location of PD-1 and PD-L1 in pNETs

PD-L1 ligand expression in pancreatic neuroendocrine neoplasms is relatively rare, observed in only approximately 7.4% of pNETs [17]. However, in the broader group of gastrointestinal and pancreatic neuroendocrine neoplasms (GEP-NENs), the frequency of positive PD-L1 expression shows a clear correlation with histological grade, increasing from 8.99% in G1 tumors, through 12.37% in G2 tumors, to 37.04% in well-differentiated G3 tumors and 48.91% in poorly differentiated G3 tumors (NECs) [51].

In pNETs, the primary source of PD-L1-expressing cells is tumor cells, but positive expression is also observed in endothelial cells and the CD45+ immune cell population present in the tumor microenvironment [52]. A study by Takahashi et al. demonstrated that the number of T lymphocytes expressing PD-1 was significantly higher in higher-grade tumors. Furthermore, T lymphocytes infiltrating pNETs were characterized by significantly higher levels of PD-1 compared to peripheral blood lymphocytes [7].

The prognostic significance of the PD-1/PD-L1 axis in pNETs is particularly relevant in the context of tumor grade. A high density of tumor-infiltrating lymphocytes (TILs) combined with high PD-1 expression correlated with shorter overall patient survival and a more aggressive tumor phenotype [43,51]. Furthermore, elevated expression of stromal PD-1 and PD-L1 was associated with a poor prognosis. The number of CD3+/PD-1high T lymphocytes and type II CD204+/PD-L1high macrophages was identified as an independent predictor of disease recurrence [7].

### 3.12. B7 Family (CD276, B7x, HHLA2)

In gastrointestinal neuroendocrine tumors, the expression of molecules belonging to the B7 family, including B7-H3 (CD276), B7x, and HHLA2, has also been intensively studied [53]. Among these, CD276 is currently one of the most promising therapeutic targets. This molecule has the ability to inhibit T cell activity, contributing to immune tumor escape. In pNENs, CD276 is particularly abundantly expressed by tumor-associated macrophages (TAMs) present in the tumor microenvironment. High levels of CD276 expression correlate with unfavorable clinicopathological features, such as lymph node involvement, high histological grade, TNM stage, distant metastasis, and shortened patient survival [39].

B7x represents another important immune surveillance point, demonstrating strong expression in human pNETs. Its activity leads to inhibition of T cell function, promoting tumor progression. B7x expression is closely associated with tumor hypoxia and HIF-1α activation, suggesting its role as a key mediator of immunosuppression in the NET microenvironment [54].

HHLA2 is another B7 family molecule whose elevated expression has been found in both pNETs and small intestinal neuroendocrine tumors. Like B7x, HHLA2 is associated with unfavorable clinical parameters and reduced T cell activity, further underscoring its importance in the pathogenesis of the immunosuppressive GEP-NEN microenvironment [54].

### 3.13. Immune Checkpoint Inhibitors—Clinical Trials

Immune checkpoint inhibitors (ICIs), particularly those targeting the PD-1/PD-L1 axis, are an intensively studied class of drugs in the treatment of neuroendocrine tumors. Despite the documented efficacy of immunotherapy in many malignancies, its role in the treatment of pancreatic neuroendocrine tumors remains limited and is not currently standard of care [43,55] (Table 1).

### 3.14. Pembrolizumab

Pembrolizumab, a humanized IgG4 monoclonal antibody directed against the PD-1 receptor, has been evaluated in several clinical trials in patients with pNENs. The Phase Ib KEYNOTE-028 study, which enrolled patients with tumors positive for PD-L1 expression, also analyzed 16 patients with pNETs. This group achieved an objective response rate (ORR) of 6.3% and a median progression-free survival (mPFS) of 4.5 months, with one patient experiencing a partial response (PR). Treatment-related adverse events (TRAEs) occurred in 69% of patients, with fatigue, diarrhea, and hypothyroidism being the most common [56].

The phase II KEYNOTE-158 study evaluated the efficacy of pembrolizumab in 107 patients with GEP-NENs who had failed prior systemic therapy, including 40 patients (37.4%) with pNETs. The ORR for the entire cohort was 3.7%, with partial response achieved only in patients with negative PD-L1 expression, including three patients with pNETs. Median overall survival (mOS) was 24.2 months, and mPFS was 4.1 months. Treatment-related adverse events occurred in 75.7% of patients, including 21.5% with grade 3–5 severity [57].

Additional data were provided by an analysis of two phase II trials (GI-087 and Moffitt-19207) conducted by Vijayvergia et al. Of the 29 patients with G3 neuroendocrine tumors, 10 had a primary tumor located in the pancreas. In this group, the ORR was 3.4% and the disease control rate (DCR) was 24.1%. No significant differences in PFS or OS were observed between patients with positive and negative PD-L1 expression [58].

Pembrolizumab was also evaluated in the phase II PLANET trial in combination with the somatostatin analog depot lanreotide. The study enrolled 22 patients with G1/2 GEP-NETs, including 8 patients with pNETs. The ORR for the entire cohort was 39%, the mPFS was 5.4 months, and the mOS was 15 months. Serious treatment-related adverse events (SAEs) occurred in only 6 patients [59].

### 3.15. Spartalizumab

Spartalizumab (PDR001), another PD-1 inhibitor, was evaluated in a multicenter phase II study involving 21 patients with poorly differentiated GEP-NECs and 95 patients with well-differentiated G1–G2 NETs, including 33 patients with pNETs. The NET group achieved an ORR of 7.4%, a 12-month PFS of 19.5%, and a 12-month OS of 73.5%, while the efficacy in the NEC group was lower. Higher response rates were observed in patients with ≥1% PD-L1 expression and ≥1% CD8+ cell infiltration, but insufficient samples were available for reliable statistical analysis [60].

### 3.16. Toripalimab

Toripalimab, a PD-1 inhibitor, was evaluated in a Phase Ib study of 40 patients with pNENs, including 9 with a primary pancreatic lesion. This cohort achieved an ORR of 22.2% and a DCR of 55.5%. Significantly better treatment outcomes were observed in patients with high PD-L1 expression and in patients with high tumor mutational burden (TMB-H). Treatment benefits were also noted in patients with microsatellite instability (MSI-H) [61].

The importance of high TMB was also confirmed in a randomized report by Cao et al., describing a patient with G3 pNET who experienced a significant increase in TMB following prior CAPTEM treatment and subsequently stabilized her disease following toripalimab [62].

### 3.17. Nivolumab and Combination Therapies

Nivolumab, a human monoclonal antibody directed against programmed cell death protein 1 (PD-1), is a well-known immune checkpoint inhibitor approved for the treatment of numerous solid tumors. Nivolumab binds specifically to PD-1 receptors, thereby blocking their interaction with PD-L1 and PD-L2, which leads to inhibition of the PD-1 pathway-dependent immune response and exerts antitumor activity [63].

The efficacy of nivolumab has been evaluated both as monotherapy and in combination therapy. In the NICE-NEC study (GETNE-T1913), the combination of nivolumab with carboplatin and etoposide in patients with G3 NENs achieved an ORR of 56.8%, an mPFS of 5.7 months, and an mOS of 13.9 months, with an acceptable toxicity profile [64].

The combination of nivolumab with temozolomide, evaluated in a phase II study by Owen et al., demonstrated an ORR of 32.1%, with a particularly long mPFS in patients with pNETs. Immunological analysis of peripheral blood indicated an increase in the percentage of CD8+ lymphocytes during treatment [65].

### 3.18. Dual Checkpoint Blockade

Combination therapy with PD-1/PD-L1 and CTLA-4 inhibitors has been studied in several clinical trials. In the DUNE study (GETNE-1601), the combination of durvalumab with tremelimumab in patients with G3–G4 pNETs demonstrated limited efficacy, with an ORR of 6.3% and an mOS of 23.8 months [66].

In the CA209-538 study, ipilimumab and nivolumab therapy in patients with advanced NETs, including pNETs, led to an ORR of 24% and a clinical benefit rate (CBR) of 72% [67]. These results were partially confirmed in a retrospective analysis by Al-Toubah et al., although the overall efficacy of the therapy remained moderate [68].

### 3.19. Combination Therapies with ICIs

ICIs have also been studied in combination with antiangiogenic drugs. The combination of atezolizumab with bevacizumab in patients with pNETs resulted in an ORR of 20% and an mPFS of 14.9 months [69]. Similarly, avelumab with regorafenib demonstrated moderate clinical activity with an acceptable safety profile [70].

### 3.20. Retrospective Analyses

A retrospective analysis by the Mayo Clinic of 57 patients with NENs confirmed the limited efficacy of ICIs monotherapy, particularly in patients with NECs. Better results were observed with combination therapies, especially with chemotherapy, but treatment effectiveness remains unsatisfactory in many NEN subtypes [71].

Taken together, the available data indicate that the role and clinical relevance of immune checkpoint pathways in pancreatic neuroendocrine neoplasms are strongly dependent on tumor differentiation and grade. Well-differentiated pNETs G1–G2 are generally characterized by low PD-L1 expression, limited immune cell infiltration, and an overall immune-cold or immune-excluded phenotype, which is consistent with the modest and inconsistent clinical activity of immune checkpoint inhibitors observed in this subgroup. In pNET G3, immune checkpoint expression and immune infiltration appear more heterogeneous, reflecting increased proliferative activity and immune pressure; in selected cases—particularly those with elevated PD-L1 expression, high tumor mutational burden, or microsatellite instability—immunotherapy may confer clinical benefit. By contrast, poorly differentiated pNECs more frequently exhibit an immune-inflamed microenvironment with higher PD-L1 expression, increased infiltration of activated immune cells, and biological features resembling other high-grade epithelial malignancies, providing a stronger mechanistic rationale for immune checkpoint blockade, particularly in combination with chemotherapy or other immunomodulatory strategies.

### 3.21. Other Therapeutic Options—Preclinical and Clinical Data

#### 3.21.1. CDK4/6 Inhibitors

Cyclin-dependent kinase 4 (CDK4) is an important regulator of the cell cycle, participating in the phosphorylation of the RB1 protein and facilitating cell progression through the G1/S checkpoint. In pNETs, its overactivation has been reported in approximately 58% of samples, and elevated CDK4 expression (similar to CDK6) correlated with a higher Ki-67 proliferation index [72].

Palbociclib (a CDK4/6 inhibitor) was evaluated in a phase II trial (PALBONET) involving 21 patients with metastatic pNETs of G1-G2. Disease stabilization was achieved in 57.9% of patients; median PFS was 2.6 months, and median OS was 18.7 months. The most frequently observed adverse events included asthenia (76.2%), neutropenia (42.9%), and diarrhea and nausea (33.3% each) [73].

#### 3.21.2. Ibrutinib

Ibrutinib, a Bruton’s kinase (BTK) inhibitor, was considered a potential therapeutic strategy, among other reasons, due to its ability to inhibit mast cell degranulation; it was associated with tumor regression in a mouse insulinoma model [74]. In a phase II study, in which 20 patients were divided into two cohorts, one of the cohorts included 5 patients (25%) with pNENs; no objective responses were observed in this subgroup (ORR 0%). The median PFS was 3 months, and the median OS was 24.1 months [75].

#### 3.21.3. Histone Deacetylase Inhibitors

Vorinostat (HDAC inhibitor) demonstrated immunomodulatory potential within the TME. In vitro, increased CCR5 expression in BON-1 and QGP-1 cell lines and CCR5-dependent recruitment of autologous T lymphocytes to freshly resected pNET tissue were observed. In the in vivo model (RIP-TVA), vorinostat treatment was associated with increased T lymphocyte infiltration (CD4+ and CD8+) in the tumor microenvironment [76].

### 3.22. Future Therapeutic Targets

#### 3.22.1. Anti-CD47 Antibodies

In preclinical studies, anti-CD47 antibodies (Hu5F9-G4, B6H12) enhanced phagocytosis of pNET cells by macrophages (human and murine) in vitro. In vivo models, they demonstrated reduced tumor growth, reduced liver metastasis, and prolonged survival in orthotopic xenograft models (APL1) and murine models [77].

#### 3.22.2. Anti-CD276 Antibodies

CD276 represents another target of growing translational importance. In pNEN samples, CD276 expression exceeded PD-L1 expression on TAM cells (78% vs. 53%) [33]. Converging results were presented by Zhong et al., indicating high expression of CD276 in all analyzed pNEN biopsies and the presence of this molecule on F4/80 macrophages in a mouse model [39]. CD276-targeted therapies, including monoclonal antibodies (e.g., enoblituzumab) and CAR-T approaches, are being studied in clinical trials for various cancers and have thus far demonstrated acceptable safety profiles [44,78]. These data suggest the validity of further evaluation of this strategy in pNENs as well.

#### 3.22.3. Combination Therapy with Anti-PD-L1 and Anti-VEGF

Combining checkpoint inhibitors (CPIs) with antiangiogenic therapy may enhance the antitumor response, including by inducing high endothelial venules (HEV) and increasing T cell infiltration of the TME [44]. In vivo experiments in mouse models (RT2-PNET, MMTV-PyMT, NFpp10-GBM), combination therapy enhanced the antitumor response [52]. In the RT2-PNET model, reduced tumor recurrence following monotherapy with a VEGF/VEGFR2 inhibitor and prolonged animal survival were demonstrated. Promising activity has also been reported in clinical trials combining immunotherapy with antiangiogenic drugs, including atezolizumab with bevacizumab [69], toripalimab with surufatinib [79], and avelumab with regorafenib [70]. Table 2.

#### 3.22.4. B7x Immune Checkpoint Inhibitor

Therapies targeting B7x remain in the preclinical stage. In vitro, pancreatic β-islet tumor cells (N134) cultured in hypoxia (1% O_2_) expressed B7x. In a mouse model of pNET, genetic elimination of B7x was associated with a reduction in tumor burden and increased T cell infiltration in the TME. Furthermore, the use of an anti-B7x antibody enhanced the T cell-dependent antitumor response (increased perforin, granzyme B, and FasL in tumor tissue), induced tumor cell apoptosis, reduced serum insulin levels, and improved animal survival [54].

#### 3.22.5. Penpulimab—A Modified Anti-PD-1 Antibody

Penpulimab remains a potential candidate for anti-PD-1 therapy in pNENs, described as an antibody with a more stable structure, slower dissociation, and reduced cytotoxicity. Li et al. presented a case report of penpulimab used in combination with a VEGF inhibitor in an 18-year-old patient with pNEC with liver metastases, achieving a PFS of 13 months without significant toxicity [80].

#### 3.22.6. CAR-T Therapy

Further development of cellular immunotherapy is considering the use of CAR-T in pNENs. In preclinical models, AdFITC(E2)-CAR T-cells, combined with the SSTR2 antagonist Octo-Fluo, effectively eliminated BON-1-SSTR2 cells in vitro, remaining inactive in the absence of target cells. In a mouse model, an antitumor effect dependent on Octo-Fluo concentration was observed [81].

#### 3.22.7. BiTE Therapy—Tidutamab

Another strategy involves BiTE (bispecific T-cell engager) antibodies, which combine a tumor antigen-binding domain with a CD3-binding domain on T lymphocytes [82]. In a non-randomized phase I study of tidutamab (XmAb^®^18087, SSTR2-directed BiTE; Manufacturer: Xencor, 465 North Halstead Street, Suite 200, Pasadena, CA, USA) involving 25 patients (including 13 with pNET), disease stabilization was achieved in 6 patients with pNET. Grade 3–4 adverse events occurred in 68% of participants; the most frequently observed were lymphopenia (44%), increased GGT (24%), ALT/AST (20%), and vomiting (20%) [72,83].

### 3.23. Independent Risk Factors for Relapse

In the context of future immunotherapeutic strategies, identifying biomarkers associated with the risk of relapse may be important. Chen et al., analyzing immunohistochemical samples from 183 patients with pNENs, identified two factors associated with the m6A (N6-methyladenosine) modification—YTHDF2 and HNRNPC—as variables associated with the risk of relapse. Additionally, HNRNPC correlated with PD-L1 expression, which may support the concept of combination therapy with anti-PD-L1 antibodies in selected patient subgroups [84].

### 3.24. VEGF Inhibitors—Clinical Data

#### 3.24.1. Bevacizumab

Bevacizumab is a humanized IgG1 monoclonal antibody targeting vascular endothelial growth factor A (VEGF-A). Beyond its direct antiangiogenic effects, inhibition of VEGF signaling has been shown to remodel the tumor microenvironment by inducing vascular normalization, improving immune cell trafficking, and modulating the recruitment and function of immunosuppressive myeloid populations, including tumor-associated macrophages [85,86]. These mechanisms are of particular relevance in pancreatic neuroendocrine tumors, which are characterized by pronounced vascularity and a macrophage-rich, immunosuppressive microenvironment.

The clinical efficacy of bevacizumab-based combinations in pNENs has been explored in several phase II studies. In a multicenter phase II trial involving 11 patients with pNENs, combination therapy with bevacizumab, pertuzumab (an anti-HER2 antibody), and octreotide LAR demonstrated limited clinical activity, with an objective response rate (ORR) of 5%, a disease control rate (DCR) of 23%, and a single partial response observed [87]. Bevacizumab was also evaluated in combination with octreotide LAR and everolimus in a randomized phase II trial enrolling 150 patients with advanced pNETs; the addition of bevacizumab did not result in a significant improvement in progression-free survival compared with regimens without bevacizumab and was associated with a higher incidence of adverse events [88] (Table 3).

Additional phase II studies investigated bevacizumab in combination with various chemotherapy regimens, including temozolomide, FOLFIRI, FOLFOX, and capecitabine. Although modest antitumor activity was observed in selected patients, none of these approaches led to a change in current therapeutic standards for pNETs [89,90,91]. Taken together, these data suggest that while bevacizumab-based regimens have not translated into clear clinical benefit in unselected pNET populations, their potential role in combination strategies aimed at tumor microenvironment modulation—particularly in conjunction with immunotherapy—remains of translational interest.

#### 3.24.2. Ziv-Aflibercept

The efficacy and safety of ziv-aflibercept, a recombinant fusion protein that neutralizes VEGF, were evaluated in a phase II study of 21 patients with advanced pNET. The ORR was 9.5% (*n* = 2), but the study was terminated prematurely due to insufficient recruitment [92].

#### 3.24.3. Sunitinib

Sunitinib, an oral tyrosine kinase inhibitor, blocks VEGFR-1, -2, and -3, PDGFR-β, and c-KIT. In a randomized phase III study of 107 patients (including 66 with pNET), sunitinib treatment was associated with an ORR of 16.7%, a one-year survival rate of 81.1%, and a median PFS of 7.7 months in the pNET subgroup [93].

In the phase II RESUNET study, which included 11 patients with metastatic pNET, sunitinib demonstrated an ORR of 9.1%, a CBR of 72.7%, and a median PFS of 7.2 months, demonstrating moderate but reproducible clinical activity [94].

#### 3.24.4. Lenvatinib

Lenvatinib, a multikinase inhibitor of VEGFR, FGFR, PDGFR-α, RET, and KIT receptors, was studied in the phase II TALENT study, which enrolled 111 patients, including 55 with pNET. This cohort demonstrated high antitumor activity, with an ORR of 44.2% and a median duration of response (DOR) of 19.9 months. The most common grade 3/4 adverse events included hypertension (22.7%), asthenia (13.6%), and diarrhea (10.9%). Dose reduction was necessary in 93.8% of patients [95].

#### 3.24.5. Pazopanib

Pazopanib, a tyrosine kinase inhibitor, was evaluated in a phase II trial in 37 patients with GEP-NETs, including 12 with primary pancreatic lesions. A DCR of 75.7% and an ORR of 18.9% were observed. The most common toxicities were proteinuria, neutropenia, and hypertension [96].

#### 3.24.6. Cabozantinib

Cabozantinib was evaluated in a randomized phase III trial comparing it with placebo in two cohorts: patients with pNETs (*n* = 95) and those with extrapancreatic NETs. In the pNET group, the median PFS was 13.8 months compared with 4.4 months in the placebo arm, and the ORR was 19% (vs. 0% in placebo). The most common grade 3/4 adverse events included hypertension (22%), fatigue (11%), and thromboembolic events (11%). Dose reduction was necessary in 68% of treated patients [97].

#### 3.24.7. Antiangiogenic Therapy—Future Goals and Challenges

One of the key clinical challenges of antiangiogenic therapy remains overcoming resistance mechanisms leading to secondary tumor revascularization and disease progression. Periostin (POSTN), an extracellular matrix protein involved in remodeling the tumor microenvironment, may play a significant role in this process. During treatment with VEGF-A inhibitors, a transient decrease in POSTN expression is observed, whereas during the resistance phase, it increases dramatically. In vivo studies using the RIP1-Tag2 mouse model demonstrated that deletion of the POSTN gene led to attenuated revascularization and significantly inhibited pNET progression during antiangiogenic therapy [98].

Interesting observations were also made in the study by Lee and co-authors, which demonstrated that inhibition of angiopoietin 2 (ANGPT2) resulted in a delay in the development of pNET liver metastases at all stages of disease progression. Importantly, both pharmacological blockade and genetic deletion of ANGPT2 were associated with prolonged survival in mouse models. The authors also noted an inverse correlation between ANGPT2 expression levels and the density of CD8+ T cell infiltration in liver metastases from pNET patients, suggesting the involvement of this factor in shaping the immunologically “cold” tumor microenvironment [99].

Another promising avenue of research is multi-targeted therapies. Schmittnaegel and co-authors described the results of studies on the bispecific antibody A2V, capable of simultaneously blocking VEGF-A and ANGPT2. A2V therapy demonstrated greater anti-tumor efficacy compared to drugs targeting single pro-angiogenic factors and led to a significant prolongation of animal survival in a mouse model, indicating the potential advantage of multi-target strategies in overcoming resistance to anti-angiogenic therapy [100].

### 3.25. Other Components of the NEN Microenvironment

In recent years, it has been increasingly recognized that the immunosuppressive nature of pNENs stems not only from the presence of immune cells with inhibitory effects, but also from the physicochemical properties of the tumor microenvironment itself. The remodeled extracellular matrix influences the architecture of the neoplastic tissue and significantly modifies the local immune response. In this context, individual ECM components and stromal cells are particularly important, as their activity in pNENs clearly promotes the creation of an environment dominated by immunosuppressive signals.

Type I collagen forms the main component of the stroma and, through its disrupted architecture, limits the influx of lymphocytes into the tumor [101]. Similarly, type IV collagen, which forms the basement membrane, hinders the migration of immune cells. Both types also act through signaling—by binding LAIR-1, they reduce the activity of CD8+ lymphocytes and support the polarization of M2 macrophages, as confirmed by correlations with markers of immunosuppressive cells [102,103]. Collagen degradation products, such as glutamine, can additionally nourish tumor cells [101]. TEM8/ANTXR1 and DDR1 activity leads to fiber reorganization, which promotes tumor cell migration and dissemination [104,105].

ECM proteoglycans modulate the immune response—SLRPGs activate antigen-presenting cells, and matrikines direct the migration of effector lymphocytes [106]. In pNETs, frequent versican expression is associated with longer DFS [107], while high endocan levels worsen the prognosis [108]. GPC3 remains unexpressed in this group of tumors [109,110]. The glycosaminoglycan profile differentiates the grades of malignancy—chondroitin predominates in the tumor stroma, and syndecan 2 is highest in well-differentiated NETs [111]. Laminin is most highly expressed in G3 NENs [112], while fibronectin participates in the formation of a premetastatic niche and may inhibit CXCL12-dependent lymphocyte migration [113,114]. Pancreatic stellate cells (PSCs) are a key element of the stroma and the basis of desmoplasia [115]. In the healthy pancreas, they remain inactive, but under the influence of TGF-β, PDGF, and oxidative stress, they transform into a myofibroblast phenotype, intensively synthesizing collagen, fibronectin, and laminin [116]. This leads to the formation of a stiff stroma that restricts the influx of T lymphocytes [117]. PSCs also secrete factors that promote the recruitment and polarization of TAMs toward M2, modulate T cell function, support the differentiation of MDSCs, and inhibit the activity of NK cells [115,116,118]. They also provide metabolites such as glutamine and alanine, facilitating cancer cell survival [116]. Adipocytes enhance immunosuppression through adipokines, which modulate the activity of lymphocytes and macrophages, favoring the predominance of M2 phenotypes. Hypoadiponectinemia deprives the TME of its anti-inflammatory effects, and abnormal leptin signaling and changes in the L/A ratio correlate with tumor aggressiveness and metastasis. As a result, adipocytes both support tumor metabolism and perpetuate an environment conducive to attenuated immune response [118,119].

## 4. Summary

The tumor microenvironment of pancreatic neuroendocrine neoplasms (pNENs) is highly heterogeneous, both in cellular composition and in physicochemical properties, and plays a central role in shaping the antitumor immune response. Importantly, this heterogeneity reflects fundamental biological differences between well-differentiated pancreatic neuroendocrine tumors (pNETs; G1–G3) and poorly differentiated pancreatic neuroendocrine carcinomas (pNECs). Based on the density and composition of immune infiltrates, pNENs can be broadly classified as immune-cold tumors, with limited immune cell infiltration, or immune-hot tumors, characterized by a more pronounced inflammatory infiltrate.

In well-differentiated pNETs, particularly G1 and G2 tumors, the tumor microenvironment is most often immune-cold or immune-excluded. In this setting, clinical outcome is closely linked to immune composition: higher densities of CD8^+^ T lymphocytes are associated with a more favorable course, whereas enrichment in regulatory T cells (Tregs) and M2-polarized macrophages promotes tumor progression and aggressive behavior [7,16,17]. Stromal components, including cancer-associated fibroblasts (CAFs), further contribute to immunosuppression by remodeling the extracellular matrix and modulating immune cell function. In addition, tumor-derived exosomes rich in immunomodulatory factors promote macrophage polarization toward an M2 phenotype, thereby enhancing invasiveness and resistance to systemic therapy [29,116].

By contrast, pNECs more frequently display features of an immune-inflamed, immune-hot microenvironment, with higher immune cell infiltration and increased expression of immune checkpoint molecules. This distinction is reflected in the activity of immune checkpoint pathways such as PD-1/PD-L1 and CTLA-4. While blockade of these pathways can restore antitumor immunity, well-differentiated pNETs generally exhibit low checkpoint expression, which likely explains the limited efficacy of immune checkpoint inhibitor monotherapy and highlights the need for combination strategies [44,67,120]. Dual immune checkpoint blockade, particularly combinations of ipilimumab and nivolumab, as well as combinations of immunotherapy with platinum-based chemotherapy or temozolomide, have shown more encouraging activity, especially in higher-grade tumors and pNECs [64,65].

At the same time, there is growing interest in patient stratification using predictive biomarkers, although the clinical relevance of PD-L1 expression alone in pNENs remains uncertain. The predominance of immune-cold phenotypes in pNETs supports the development of therapeutic approaches aimed at remodeling the tumor microenvironment, including modulation of macrophage polarization and inhibition of pathways that promote regulatory T-cell activity [34].

Despite its potential prognostic value, heterogeneous testing materials (primary and metastatic) and unknown interactions with other immunological biomarkers limit clinical interpretation. TMB is a predictive factor in solid tumors. The KEYNOTE-158 clinical trial, which included patients with NENs, led to the approval of pembrolizumab for patients with TMB ≥ 10 mut/Mb, regardless of primary tumor location. The Job van Riet study reported a lower TMB of 1.09 mut/Mb in NENs compared with 5.45 mut/Mb in NECs. Like PD-L1 expression, TMB is not a perfect predictive biomarker of immunotherapy efficacy. Possible explanations include the “quality” rather than the quantity of somatic mutations. Furthermore, both are further elements of the NEN immune environment [121].

Antiangiogenic therapies show limited efficacy as monotherapy; however, combinations with chemotherapy—particularly bevacizumab with temozolomide or capecitabine—have yielded more favorable results, whereas combinations with FOLFIRI have proven ineffective [65,91]. Among tyrosine kinase inhibitors, sunitinib remains the most extensively validated option, while surufatinib, pazopanib, cabozantinib, sorafenib, and especially lenvatinib have also demonstrated clinical activity in pNETs [93,96,98]. Notably, antiangiogenic agents may further enhance responsiveness to immunotherapy by modulating the tumor microenvironment and increasing tumor immunogenicity, providing a rationale for integrated, biology-driven treatment strategies [52].

## Figures and Tables

**Table 1:** Research on the use of immunotherapy in the treatment of NETs.

Drug	NCT	Study Model	Phase	ORR [%]	mPFS [Months]	mOS [Months]	Number of Patients with pNEN
Pembrolizumab	NCT02054806	nonrandomized, single-arm, open-label	1b	6.3	4.5	NA	16
Pembrolizumab	NCT02628067	nonrandomized, parallel assignment, open-label	2	3.7 *	4.1 *	24.2 *	40
Pembrolizumab	NCT02939651	nonrandomized, single-arm, open-label	2	3.4 *	8.9 *	20.4 *	10
Pembrolizumab + Lanreotyd	NCT03043664	nonrandomized, single-arm, open-label	2	39 *	5.4 *	15 *	8
Spartalizumab	NCT02955069	nonrandomized, single-arm, open-label	2	7.4 *	3.8 *	23.4 *	12
Toripalimab	NCT03167853	nonrandomized, single-arm, open-label	1b	22.2	2.5 *	7.8 *	4
Avelumab	NCT03278405, NCT03278379	nonrandomized, single-arm, open-label	1, 2	0 *	3.3 *	14.2 *	9
Nivolumab + Carboplatin + Etoposid	NCT03980925	nonrandomized, single-arm, open-label	2	54.1 *	5.7 *	13.9 *	15
Nivolumab + Temozolomide	NCT03728361	nonrandomized, single-arm, open-label	2	35.7 *	8,8 *	32.3 *	3
Durvalumab + Tremelimumab	NCT03095274	nonrandomized, single-arm, open-label	2	6.3	5.5	23.8	32
Ipilimumab + Nivolumab	NCT02834013	nonrandomized, parallel assignment, open-label	2	11	3	24	19
Atezolizumab + Bevacizumab	NCT03074513	nonrandomized, single-arm, open-label	2	20	14.9	30.1	20
Topiralimab + Surufatinib	NCT03879057	nonrandomized, parallel assignment, open-label	1	24.1 *	4 *	NA	4

* results for the entire kohort of patients regardless of type of tumor. NA Not Available.

**Table 2:** Current clinical trials enrolling patients with pNET.

Drug	NCT	Study Model	Phase	Primary Endopoints	Inclusion Criteria
**Dostarlimab**	NCT06333314	randomized, parallel assignment, open-label	2	PFS	dMMR/MSI tumors
**IL13Ralpha2 CAR-T cells**	NCT04119024	nonrandomized, single-arm, open-label	1	Incidence of AEs, DLT	Unresectable malignancy
**Sunitinib + Lu177 Dotatate**	NCT05687123	nonrandomized, single-arm, open-label	1	Incidence of AEs	Metastatic, unresectable pNET
**Surufatinib**	NCT06158516	randomized, parallel assignment, quadruple-masking	2	2-year DFS	pNET
**Sorafenib + Gefitinib**	NCT06592989	observational, prospective	2	ORR, PFS	pNEN with progression after previous treatment
**Belzutifan**	NCT04924075	nonrandomized, single-arm, open-label	2	ORR	Advanced/metastatic tumors, including pNET

**Table 3:** Research on the use of anti-angiogenic drugs in the treatment of NETs.

Drug	NCT	Study Model	Phase	ORR [%]	mPFS [Months]	mOS [Months]	Number of Patients with pNEN
Bevacizumab + Pertuzumab + Octreotide	NCT01121939	nonrandomized, single-arm, open-label	2	5	5.49	26.4	11
Octreotide LAR + Everolimus ± Bevacizumab	NCT01229943	randomized, parallel assignment, open-label	2	-	16.7	42.5	150
Bevacizumab + Temozolomide	NCT00137774	nonrandomized, single-arm, open-label	2	33	14.3	41.7	15
Bevacizumab + Octreotide LAR + Capecitabine	NCT01203306	nonrandomized, single-arm, open-label	2	26.3	14.9	-	19
Bevacizumab + FOLFIRI	NCT02820857	randomized, parallel assignment, open-label	2	25 *	-	-	34
Capecitabine + Temzolomide + Bevacizumab	NCT01525082	nonrandomized, single-arm, open-label	2	47.4	25.2	49.8	19
Bevacizumab + FOLFOX	NCT00227617	nonrandomized, single-arm, open-label	2/3	-	-	31 *	12
Ziv-Afilbercept	NCT02101918	nonrandomized, single-arm, open-label	2	9.5	-	-	21
Sunitinib	NCT00428597	randomized, parallel assignment, double-blind	3	16.7	-	-	86
Sunitinib	NCT01121562	nonrandomized, single-arm, open-label	2	50	-	-	12
Sunitinib	NCT00428597	randomized, parallel assignment, double-blind	3	9.3	11.4	20.6 to NA	171
Sunitinib	NCT01525550	nonrandomized, single-arm, open-label	4	24.5	13.2	37.9 ** NA ***	106
Sunitinib + Evofosfamide	NCT02402062	nonrandomized, single-arm, open-label	2	17.6	10.38	32.32	17
Sulfatinib	NCT02589821	randomized, placebo-controlled, double-blind	3	19	10.9	-	172
Lenvatinib	NCT02678780	randomized, parallel assignment, open-label	2	44.2	15.6	32	55
Pazopanib	NCT01099540	nonrandomized, single-arm, open-label	2	18.9 *	9.1 *	-	12
Pazopanib + Octreotide depot	NCT00454363	nonrandomized, single-arm, open-label	2	21.9	14.2	25	32
Cabozantinib	NCT03375320	randomized, parallel assignment, double blind	3	19	13.8	40	95

* results for the entire kohort of patients regardless of type of tumor; **—Sunitinib: Later-Line Cohort; *** Sunitinib: Treatment Naive Cohort. NA Not Available.

## Data Availability

No new data were created or analyzed in this study.
